# A Lipopeptide Facilitate Induction of *Mycobacterium leprae* Killing in Host Cells

**DOI:** 10.1371/journal.pntd.0001401

**Published:** 2011-11-22

**Authors:** Yumi Maeda, Toshiki Tamura, Yasuo Fukutomi, Tetsu Mukai, Masanori Kai, Masahiko Makino

**Affiliations:** Department of Mycobacteriology, Leprosy Research Center, National Institute of Infectious Diseases, Tokyo, Japan; Institute of Tropical Medicine (NEKKEN), Japan

## Abstract

Little is known of the direct microbicidal activity of T cells in leprosy, so a lipopeptide consisting of the N-terminal 13 amino acids lipopeptide (LipoK) of a 33-kD lipoprotein of *Mycobacterium leprae*, was synthesized. LipoK activated *M. leprae* infected human dendritic cells (DCs) to induce the production of IL-12. These activated DCs stimulated autologous CD4^+^ or CD8^+^ T cells towards type 1 immune response by inducing interferon-gamma secretion. T cell proliferation was also evident from the CFSE labeling of target CD4^+^ or CD8^+^ T cells. The direct microbicidal activity of T cells in the control of *M. leprae* multiplication is not well understood. The present study showed significant production of granulysin, granzyme B and perforin from these activated CD4^+^ and CD8^+^ T cells when stimulated with LipoK activated, *M. leprae* infected DCs. Assessment of the viability of *M. leprae* in DCs indicated LipoK mediated T cell-dependent killing of *M. leprae*. Remarkably, granulysin as well as granzyme B could directly kill *M. leprae in vitro*. Our results provide evidence that LipoK could facilitate *M. leprae* killing through the production of effector molecules granulysin and granzyme B in T cells.

## Author Summary

We observed that LipoK (*Mycobacterium leprae* lipopeptide with 13 amino acids) is capable of inducing a good immune response in *M. leprae* infected human dendritic cells (DCs). These activated DCs had up-regulated expression of costimulatory molecule CD86 as well as CD83 (well known maturation marker) on their surface, and secreted IL-12, which is an important cytokine involved in the host defense against pathogens. Importantly, these mature DCs were capable of further driving type 1 responses by stimulating CD4^+^ T cells and CD8^+^ T cells for proliferation and interferon-gamma production. Further, both subsets of T cells were capable of producing cytotoxic granules: granulysin and granzyme B. *In vitro* experiments proved that these molecules are capable of killing *M. leprae* directly. It is the first report of the type, which proves that granulysin as well as granzyme B could partially kill *M. leprae*. LipoK would facilitate in inducing the immune responses in patients' harboring *M. leprae*.

## Introduction

The introduction of multidrug therapy in 1982 and the WHO campaign for the ‘elimination of leprosy as a public health problem’, have contributed greatly to the decrease in the prevalence rate over the past three decades. But leprosy still remains to be a public health problem in some countries, and the number of new cases detected during the last three years, remain steady [1]. The disease presents as a clinical spectrum that correlates with the level of the immune response to the pathogen [2]. Patients with lepromatous form of the disease have poor cellular immunity, resulting in extensive intracellular proliferation of *Mycobacterium leprae* bacilli in the skin and nerves. On the other hand, patients with the tuberculoid form of the disease are relatively resistant to the bacilli, so that few, if any, demonstrable bacilli are seen in the lesions [2], [3]. For patients with abundant bacilli, whose lesions are characterized by type-2 cytokines, there is a need to up-regulate the T-cell mediated type 1 immune responses, by immunotherapeutic means to kill the bacilli.

We have previously identified a lipoprotein of *M. leprae*, a 33-kD lipoprotein (ML0603) [4]. Truncated protein, having the N-terminal 60 amino acids of 33-kD lipoprotein, had cytokine inducing ability in human monocytes, in contrast to the C-terminal 192 amino acids having no such ability [5]. In this study, we synthesized the lipopeptide (LipoK) having the N-terminal 13 amino acids of the 33-kD *M. leprae* lipoprotein linked to tri-palmitoylated portion of a lipid. Since GC mass spectrometry of mycobacterial lipoproteins provided evidence for the presence of three fatty acids (either palmitic, stearic or tuberculostearic acid), we assumed that tri-palmitoylated peptide would represent the natural lipoprotein of *M. leprae* [6], [7]. Further, N-acyl transferase (Lnt) activity was identified in mycobacteria, which transfers the amide-linked acyl group to the N-terminal cysteine residue [6]. This presence of Lnt activity would indicate the presence of triacylated lipoproteins in mycobacteria, although the exact lipid structure of *M. leprae* lipoprotein is still to be determined. Previously, it was observed that hexameric peptides with tri-palmitoyl modification, corresponding to 19-kD and 33-kD lipoproteins of *M. leprae*, partially activates cells through TLR2-TLR1 heterodimers [8], [9]. Since dendritic cells (DCs) are the most potent antigen presenting cells capable of bacilli uptake, antigen presentation and initiating acquired immune responses, DCs were used as target antigen presenting cells, in the present study [10], [11]. As expected, it was found that LipoK, delivered signals through TLR2, and activated *M. leprae* infected DCs to produce abundant IL-12, although, LipoK does not produce IL-12, in non-infected DCs. Several mechanisms are known to be involved in the clearance of intracellular bacteria, including interferon gamma (IFN-γ) release, apoptosis induction of the host cells and anti-microbial activity of CD8^+^ cytotoxic T lymphocytes (CTL) [12]–[15]. CTL mediated killing of mycobacteria, was demonstrated in tuberculosis by Thoma-Uszynski *et al.* They showed that CD8^+^ CTL-mediated killing of *M. tuberculosis* was dependent on granule exocytosis [16].

In the present study, we analyzed whether *M. leprae* infected DCs, activated through LipoK could undergo functional changes and subsequently induce type 1 T cell activation to kill the bacilli. We observed that LipoK is a potent inducer of T cells equipped with cytolytic function, which can largely contribute to the killing of *M. leprae* in host cells.

## Materials and Methods

### Ethics statement, cell culture and preparation of the bacteria

Peripheral blood was obtained from healthy Japanese individuals under informed consent. But no information of the donor (exposure to bacilli) was provided. In Japan, BCG vaccination is compulsory for children (0∼4 years old). Monocyte-derived DCs were differentiated from monocytes using GM-CSF and IL-4 as described earlier [17], [18]. Animal studies were carried out in strict accordance with the recommendations from Japan's Animal Protection Law. The protocol was approved by the Experimental Animal Committee, of the National Institute of Infectious Diseases, Tokyo (Permit Number: 210001). *M. leprae* (Thai-53 strain) is passaged in athymic *nu/nu* mice (Clea Co, Tokyo) [19]. At 8 to 9 months post-infection, the footpads were processed to recover *M. leprae* [20]. For all experiments, *M. leprae* was freshly prepared. The multiplicity of infection (MOI) was determined based on the assumption that DCs were equally susceptible to infection with *M. leprae* [21], and immature DCs were infected with *M. leprae* at MOI 50 in all experiments. Human cells without the bacilli was cultured at 37°C, but when infected with the bacilli, the cells were cultured at 35°C, which is the minimal temperature at which the cells can survive in *in-vitro* experiments. LipoK having the structure Palmitoyl-Cys((RS)-2,3-di(palmitoyloxy) -propyl)-Leu-Pro-Asp-Trp-Leu-Ser-Gly-Phe-Leu-Thr-Gly-Gly-OH, was synthesized by Bachem (Bubendorf, Switzerland). Using LAL assay (QCL-1000, Lonza), endotoxin was undetectable in original LipoK preparation (50 µg/ml). Therefore, any contaminating LPS in the synthesized product could be ruled out. Monoclonal Ab to TLR2 was kindly provided by Genentech, and mAb to mannose receptor and DC-SIGN were obtained from BD Biosciences. Parthenolide obtained from Santa-Cruz was used at a concentration of 2 and 5 µM. CD40L (Pepro Tech) was used at the concentration of 1 µg/ml, whenever needed.

### Analysis of cell surface Ags on DCs and measurement of IL-12 production

Immature DCs were stimulated with *M. leprae* and/or LipoK for 48 hours. The expression of cell surface antigens on DCs, were analyzed using FACSCalibur flow cytometer (BD Biosciences). Dead cells were eliminated from the analysis by staining with 7-amino actinomycin D stain. For analysis of cell surface Ag, the following mAb were used: FITC-conjugated mAb against HLA-ABC (G46-2.6), HLA-DR (L243) and CD86 (FUN-1), purchased from PharMingen, and CD83 (HB15a, Immunotech). The ability of DCs to produce IL-12 on stimulation with either LipoK and/or *M. leprae*, was assessed. DCs were stimulated with the Ags on day 4 after the start of culture from monocytes. After 24 hours, OptEIA Human IL-12 (p70) ELISA Set (BD Biosciences) was used to determine the concentration of IL-12 p70 in the culture supernatant.

### DC-T cell co-cultures

The ability of *M. leprae*-infected DCs to stimulate T cells was assessed using an autologous DC-T cell co-culture. CD4^+^ T cells and CD8^+^ T cells were purified using respective T cell enrichment Set (BD IMag) from freshly thawed PBMCs. The purity of CD4^+^/CD8^+^T cells was determined to be more than 95%. The purified responder cells (1×10^5^ per well) were plated in 96-well round-bottom tissue culture plates, and mitomycin C-treated DCs which were pulsed with Ag, were added to give the indicated DC: CD4^+^ or CD8^+^ T cell ratio. Supernatants of DC-T cell co-cultures were collected on day 4, and IFN-γ production was measured by ELISA, using Opt EIA Human IFN-γ ELISA Set (BD Biosciences). In other experiments, Ag-pulsed DCs were treated with mAb to HLA-ABC (W6/32), HLA-DR (L243), CD86 (IT2.2) or normal mouse IgG. For obtaining naïve T cells, anti-CD45RO mAb (Dako) and anti-mouse IgG Ab Dynabeads M-450 (Invitrogen) were used to negatively select the cells. Since BCG is compulsory for children in Japan, it is likely that naïve T cells respond to *M. leprae* antigens, some of which are cross reactive to *M. bovis* BCG.

### Measurement of T cell proliferation by CFSE labeling

DCs stimulated with Ags were co-cultured with the CFSE labeled total T cells. CFSE (Molecular Probes) was added at the concentration of 1 µM and incubated at 37°C for 10 min and stabilized according to the manufacturers' protocol. A total of 1×10^6^ cells/well were seeded in a 24-well plate at a DC∶T cell ratio of 1∶6. After 8 days co-culture, cells were co-stained with PE conjugated anti-CD4 mAb and APC conjugated anti-CD8 mAb (BD Biosciences). CFSE signal of gated T cells were analysed.

### Confocal microscopy

Imaging of cells was performed using laser scanning microscope LSM5-Exciter (Carl Zeiss). DCs grown on a 13-mm coverglass in a 24-well plate, were infected with *M. leprae* and/or stimulated with LipoK for 48 hours. T cell from the same donor was purified using the Dynal T cell isolation kit, and co-cultured with DCs for additional 3 days, after washing out extracellular bacilli. Cells were fixed in 2% paraformaldehyde, and the bacilli stained with 0.01% auramine O as described [22]. Anti-*M. leprae* membrane (minus LAM) polyclonal antibody was kindly provided by Dr. John S. Spencer through the NIH/NIAID Leprosy Research Support (N01 A1-25469). Fixed cells were blocked with normal human IgG (10 µg/ml), and stained with the above polyclonal antibody (1 µg/ml) for 30 min in PBS containing 0.1% saponin and 0.5% BSA, and the secondary antibody used was Alexa Fluor 633-conjugated goat anti-rabbit IgG (Molecular Probes), and images were recorded on fluorescent confocal microscope using a 63× oil objective, 488-nm and 633-nm lasers. Data was processed using the LSM software ZEN 2007. All bacilli observed were not surface attached as observed by section scanning (Z-stack Navigation).

### Determination of intracellular levels of perforin, granzyme B and granulysin

After 7 days co-culture of purified T cells with DC pulsed with *M. leprae* and/or LipoK, intracellular detection of cytolytic effector molecules was performed. Briefly, GolgiStop (BD Biosciences) was added to the media for the last 12 hours of culture. Cells were first surface stained, fixed, permeabilized, and finally stained with FITC conjugated anti-perforin mAb or anti-granzyme B mAb or isotype control IgG2a (BD Biosciences). For the determination of intracellular levels of granulysin, the procedure was followed as for the intracellular stain of perforin, except that the surface stain used was FITC conjugated-CD4 and APC conjugated anti-CD8 mAb (BD Biosciences), and subsequently PE conjugated granulysin (eBioscience, GmbH, Germany) was used to determine the percentage of granulysin producing cells.

### Determination of *M. leprae* viability in DCs

Since *M. leprae* cannot be cultured in vitro, we measured the viability of the bacilli, by the measurement of radioactive CO_2_ production from oxidation of palmitic acid as described previously [23]. DCs were infected with *M. leprae* with or without LipoK, and co-cultured with T cells in some cases. Six days later, cells were harvested and washed 3 times in PBS, and centrifuged, so that *M. leprae* that might have escaped from the DCs into the media could be eliminated from our assay. Cell lysates were prepared as follows: 0.1 N NaOH solution was added to the cells for few minutes and then neutralized with the equal volumes of 0.1 N HCl solution. Subsequently, equal volume of 2 times concentrated Middlebrook 7H9 broth supplemented with ADC was added. ^14^C labeled palmitic acid was added to the lysates of DCs and cultured at 33°C. Seven days later, the amount of ^14^CO_2_ evolved and trapped on the filter paper was measured using a Packard 1500 TRI-CARB liquid scintillation analyzer. In a likewise manner, direct effect of *M. leprae* killing was observed by incubation of the bacilli with 3 µg/ml of granulysin (R&D systems) or granzyme B (Calbiochem) for a period of 3 days at 33°C, and then ^14^C labeled palmitic acid was added to determine the viability as described above.

### Statistical analysis

The unpaired student's t test was used to find the significance of the two sets of data. Differences were considered as statistically significant if p<0.05. All experiments were performed at least 3 times with different blood donors, unless otherwise stated, and the reproducibility of the experiment was evaluated. In some cases, ANOVA was used for probability calculation.

## Results

### LipoK activated human dendritic cells

**Figure 1:**
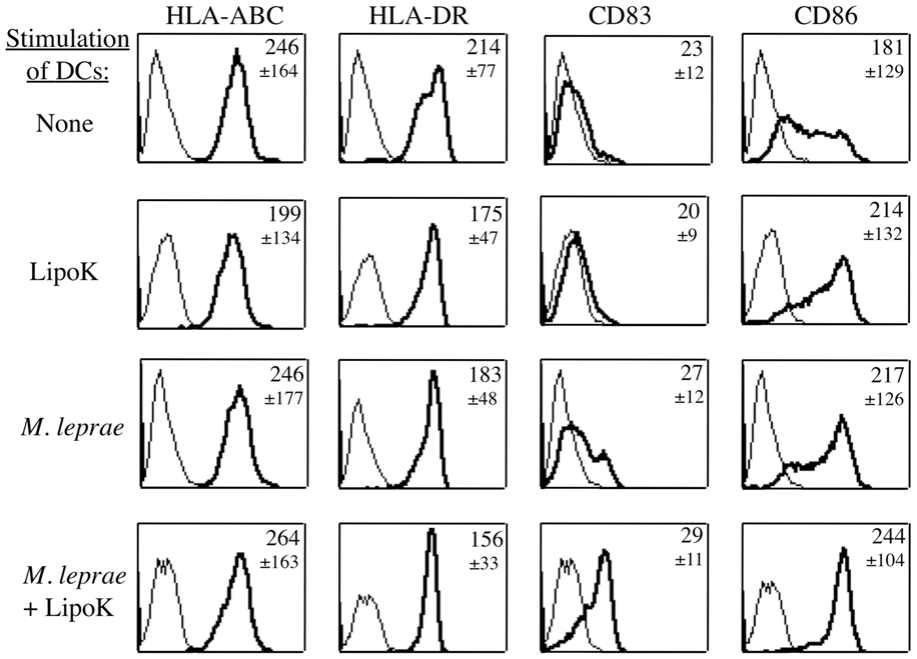
Expression of the surface markers on DCs after stimulation of *M. leprae* infected DCs with LipoK. The expression of cell surface markers on DCs, was analyzed using FACSCalibur. Dead cells were eliminated from the analysis by staining with 7-amino actinomycin D (7-AAD) stain. LipoK was used at a concentration of 0.3 µg/ml. The following mAb were used: FITC-conjugated mAb against HLA-ABC, HLA-DR, CD83 and CD86. Black light lines, isotype-matched control IgG. Black solid lines show the fluorescence intensity of the respective surface markers of DCs. Numbers indicate the mean fluorescence intensity with SD of the respective surface markers. Representative data of three separate experiments with different donors is shown.

We investigated the effect of LipoK stimulation on human monocyte derived DCs. All DCs were CD1a positive and CD14 negative [21]. When LipoK was used as a stimulant for immature DCs, maturation of DCs was observed as shown in Fig. 1. Up-regulation in the expression of CD83 (maturation marker of DCs) and CD86 (co-stimulatory molecule) was observed in LipoK stimulated DCs, the level of which, was similar to that of *M. leprae* infected DCs. *M. leprae* was used at the multiplicity of infection (MOI): 50 in all the experiments. The expression of the CD83 and CD86 molecules was more pronounced when LipoK was used to stimulate *M. leprae* infected DCs. The expression of HLA-ABC and HLA-DR molecules was not significantly different in LipoK stimulated *M. leprae* infected DCs from non-infected DCs, after 48 hours. Although, at earlier time points (18 hours after stimulation with antigen), a higher expression of HLA-ABC and HLA-DR is observed in LipoK stimulated *M. leprae* infected DCs compared to non-stimulated.

**Figure 2:**
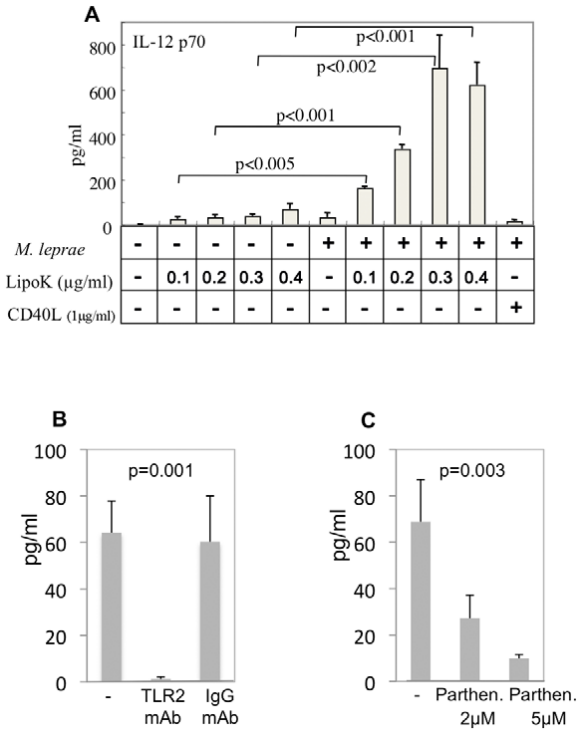
Production of IL-12 p70 from DCs. (**A**) Enhanced induction of IL-12 p70 from DCs by stimulation with LipoK and *M. leprae*. DCs were stimulated with the antigens on day 4 after the start of culture from monocytes. After 24 hours, IL-12p70 concentration in the culture supernatant was measured by the enzyme assay kit Opt EIA Human ELISA Set. The antigens used for the stimulation were: *M. leprae* and LipoK at different concentrations 0.1 µg/ml, 0.2 µg/ml, 0.3 µg/ml, and 0.4 µg/ml, CD40L was used at 1 µg/ml. (**B**) IL-12 p70 production from LipoK stimulated *M. leprae* infected DCs, is inhibited by antagonistic antibodies to TLR-2, and not by control normal IgG. (**C**) Effect of 2 µM and 5 µM of parthenolide (parthen.) on the IL-12 production was observed. Representative data of three separate experiments with different donors is shown. The probability by ANOVA was calculated to be 0.001 for (B) and 0.003 for (C).

Alternatively, when the IL-12 p70 secreted by DCs was measured, increasing dose of LipoK on *M. leprae* infected DCs produced the cytokine, with maximal cytokine production at LipoK concentration of 0.3 µg/ml (Fig. 2A). LipoK alone did not produce statistically significant amounts of IL-12 at the concentration of 0.3 µg/ml compared to the non-stimulated DCs. Another TLR-2 agonist, peptidoglycan could produce IL-12 (data not shown), probably due to the heterogeneous nature of the peptidoglycan which contains long peptide linkages. LipoK probably need other protein/peptide molecules to activate IL-12 production in DCs. Also, *M. leprae* infection alone did not produce IL-12 in DCs. When CD40 ligand (CD40L) was used to stimulate *M. leprae* infected DCs, IL-12 production was negligible.

As could be expected, TLR-2 antagonistic Ab completely blocked IL-12 production, whereas mannose receptor Ab did not, suggesting that IL-12 production from LipoK stimulated *M. leprae* infected DCs was TLR-2 dependent (Fig. 2B). When DCs were pre-treated with parthenolide, which is known to inhibit NF-kB activity [24], it was found that both 2 µM and 5 µM could significantly inhibit the production of IL-12 in a dose-dependent manner (Fig. 2C), indicating that NF-kB is involved in the IL-12 production from these LipoK stimulated DCs.

### LipoK pulsed human DCs activated human T cells *ex vivo*

**Figure 3:**
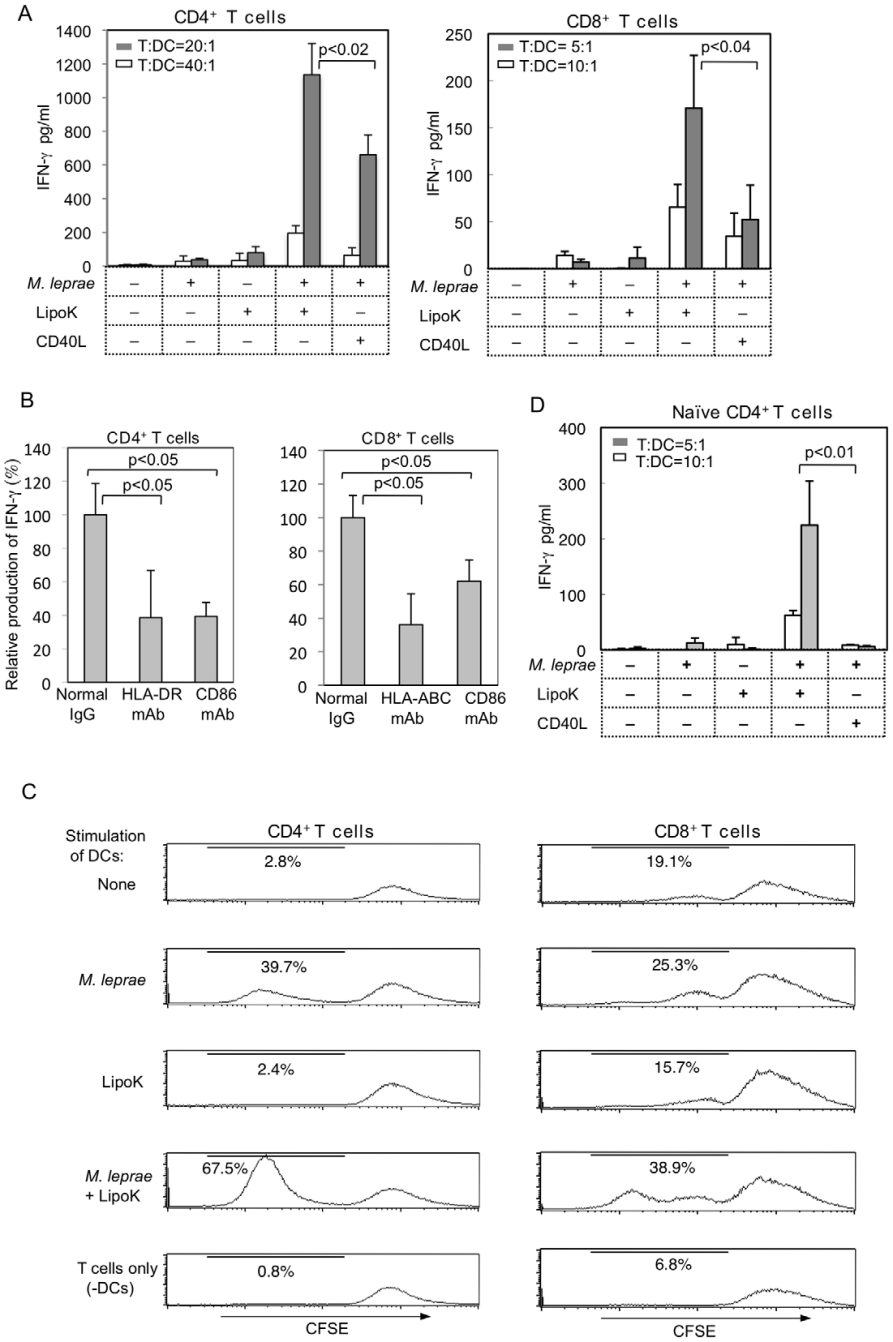
T cell activity as determined IFN-γ production and T cell proliferation. (**A**) The effect of LipoK on *M. leprae*-infected DCs to stimulate T cells was assessed using an autologous DC-CD4^+^ or DC-CD8^+^ T cell co-culture. IFN-γ production in the supernatant was measured by ELISA, after 4 days co-culture. (**B**) Effect of normal murine IgG or mAb to HLA-ABC/HLA-DR or CD86 on IFN-γ production from T cells co-cultured with *M. leprae* infected DCs simulated with LipoK. The production of IFN-γ from Ab non-treated T cells, cultured with LipoK and *M. leprae* stimulated DCs, is considered 100% and the actual value of IFN-γ produced from CD4^+^ T cells is 250 pg/ml and that from CD8^+^ T cells is 47 pg/ml at T cell∶DC ratio of 10∶1. (**C**) Proliferation of CD4^+^ and CD8^+^ T cells as assessed by CFSE labeling of T cells. DCs were mixed with autologous CFSE labeled T cells at a T cell∶DC ratio of 10∶1. Proliferating T cells were analysed by FACSCalibur on day 7 after co-culture. The percentage of proliferated cells is indicated. The lowest histogram shows unstimulated T cells. (**D**) IFN-γ production from DC-naïve CD4^+^ T cell co-culture. IFN-γ production was measured after 4 days co-culture with stimulated DCs. Representative data of four separate experiments with different donors is shown. Assays were performed in triplicate and the results are expressed as the mean +SD.

To investigate the effect of LipoK on T cell responses, purified CD4^+^ and CD8^+^ T cells from autologous donors were cultured with activated DCs. IFN-γ release was measured as correlates of T cell activation. When the IFN-γ levels were compared, DCs activated with *M. leprae* and LipoK produced significantly higher dose of IFN-γ from CD4^+^ T cells, when compared to that produced by DCs stimulated with *M. leprae* or LipoK alone, or *M. leprae* in presence of CD40L (Fig. 3A), at both high (T∶DC = 20∶1) and low (T∶DC = 40∶1) dose of DCs. Note that *M. leprae*-infection or LipoK-stimulation alone was not efficient in stimulating T cells. Similarly, secretion of IFN-γ was also observed from CD8^+^ T cells but at lower level compared to that from CD4^+^ T cells. Again there was significantly high production of IFN-γ from CD8^+^ T cells co-cultured with LipoK stimulated *M. leprae*-infected DCs compared to that from CD40L stimulated *M. leprae*-infected DCs (Fig. 3A). Although the IL-12 p70 production differed in LipoK stimulated *M. leprae*-infected DCs and CD40L stimulated DCs, no IL-12 production was observed from these mitomycin treated DCs which were co-cultured with T cells. In addition, as shown in Fig. 3B, although normal murine IgG did not affect the T cell stimulating activity of both CD4^+^ and CD8^+^ T cells, mAbs to HLA-ABC and HLA-DR, inhibited CD8^+^ T cells and CD4^+^ T cell activation of LipoK-stimulated *M. leprae*-infected DCs' respectively. The results indicated that the activation of these T cells were MHC Class II- and Class I-dependent in CD4^+^ T cell and CD8^+^ T cells respectively. The inhibition was comparable to that of inhibition of IFN-γ production by mAb to co-stimulatory molecule CD86.

Proliferation of these LipoK activated CD4^+^ and CD8^+^ T cells, was confirmed by the CFSE labeling of T cells. The labeling experiment was preferable because it could measure proliferation of individual T cell subsets even in the presence of the other subsets. *M. leprae* stimulation of DCs resulted in proliferation of 39.7% of total CD4^+^ T cells, but stimulation with both LipoK and *M. leprae* resulted in proliferation of 67.5% of total CD4^+^ T cells. LipoK stimulation alone did not induce any significant proliferation of CD4^+^ T cells (Fig. 3C). The profiles of flow cytometric analyses showed that 25.3% of CD8^+^ T cells proliferated by stimulation with *M. leprae* alone, but higher number of cells proliferated (38.9%) in presence of LipoK stimulus.

Subsequently, we examined the response of naïve T cells to LipoK activated DCs. When naïve CD4^+^ T cells were cultured with DCs activated with *M. leprae* and LipoK, significantly higher dose of IFN-γ was produced in comparison to those cultured with DCs stimulated with *M. leprae* alone or LipoK alone. Production of IFN-γ was low from those activated with *M. leprae* and CD40L (Fig. 3D). It was observed that the IFN-γ production from naïve CD8^+^ T cells, co-cultured with DCs stimulated with *M. leprae* and LipoK was meager.

When *M. bovis BCG* was used for infecting DCs, the MOI of the bacilli had to be lowered to almost 1∼10, because higher MOI (50) would kill the DCs in *in-vitro* culture. BCG when infected at MOI:1 produced 156 pg/ml of IFN-γ from CD8 T cells, but when LipoK was used to stimulate BCG infected DCs, the amount of IFN-γ increased to 380 pg/ml, indicating that LipoK could lead to further T cell activation of BCG infected DCs. It is also likely that LipoK stimulation could increase the production of perforin and granulysin in *M. tuberculosis* infected host cells.

### Up-regulation of perforin, granzyme B and granulysin production in CD4^+^ and CD8^+^ T cells

**Figure 4:**
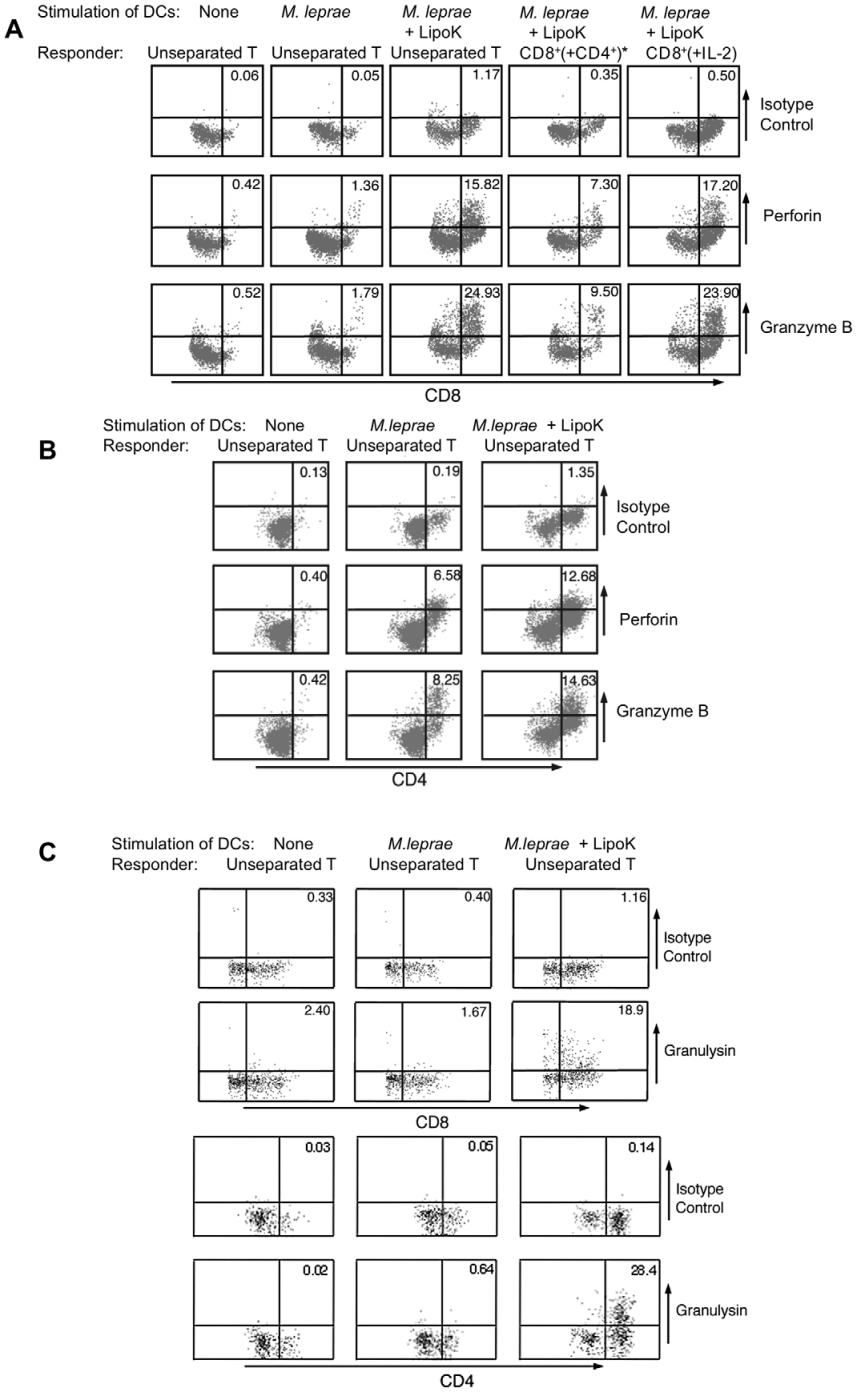
Production of perforin, granzyme B and granulysin in CD8^+^ T cells as well as CD4^+^ T cells. (**A**) Enhanced production of perforin and granzyme B from CD8^+^ T cells cultured with LipoK stimulated *M. leprae* infected DCs. Intracellular staining of perforin and granzyme B was performed as follows: Cells were first stained with PE conjugated anti-CD4 or APC conjugated anti-CD8 mAb. Then, the cells were fixed in 2% formaldehyde, permeabilized in 0.1% saponin, and stained with FITC conjugated anti-perforin mAb or anti-granzyme B mAb or isotype control IgG2a. Figure shows the dot plot of the gated CD8^+^ T cells. The right hand quadrant shows CD8^high^ T cells (activated CD8^+^ T cells) and the number indicates the percentage of perforin or granzyme B positive T cells among gated CD8^high^ T cells. *To determine whether direct interaction between CD4^+^ and CD8^+^ T cells for perforin and granzyme B production from CD8^+^ T cells, is needed, CD4^+^ T cells were cultured in inserts in a 24-well plate, and were not allowed to interact directly with CD8^+^ T cells. As a control experiment, exogenous IL-2 (in the left hand dot plot) at a concentration of 50 U/ml was added to CD8^+^ T cells. (**B**) Enhanced expression of perforin and granzyme B from CD4^+^ T cells. The right hand quadrant shows CD4^high^ T cells, and the number indicates percentage of CD4^high^ T cells producing perforin and granzyme B. (**C**) Enhanced expression of granulysin from CD8^+^ and CD4^+^ T cells, co-cultured with LipoK and *M. leprae* stimulated DCs. The protocol was followed as per the staining of perforin, except that the surface stain used was FITC conjugated-CD4 and APC conjugated anti-CD8 mAb, and subsequently PE conjugated granulysin was used. Figure shows the dot plot of the gated CD8^+^ and CD4^+^ T cells. The right hand quadrant shows CD8^high^ or CD4^high^ T cells (activated T cells) and the number indicates the percentage of granulysin positive T cells among gated CD8^high^ and CD4^high^ T cells. Representative data of three separate experiments with different donors is shown.

To determine whether cytotoxic effect could be induced in highly activated T cells, we analysed the intracellular production of perforin and granzyme B in DC co-culture system with unseparated T cells. As seen in Fig. 4A, 15.8% of activated CD8^high^ T cells produced perforin and 24.9% produced granzyme B when stimulated with DCs activated with *M. leprae* and LipoK, in comparison to those co-cultured with DCs activated with *M. leprae*, showing 1.4% of perforin and 1.8% of granzyme B-producing T cells. Thus, prominent enhancement of both perforin and granzyme B producing CD8^+^ T cells was observed. Recently, since CD4^+^ T cells are also known to possess direct cytotoxic potential [25], we measured the percentage of CD4^+^ T cells producing perforin and granzyme B. When LipoK and *M. leprae* stimulated DCs were co-cultured with T cells, 12.7% of CD4^high^ T cells produced perforin and 14.6% of those cells produced granzyme B, whereas in presence of *M. leprae* stimulated DCs, 6.6% produced perforin and 8.3% produced granzyme B (Fig. 4B). These data indicated that in addition to CD8^+^ T cells, CD4^+^ T cells also had the capacity to produce significant amounts of perforin and granzyme B. Nevertheless, the percentage of CD8^+^ T cells producing these cytolytic proteins was 1.2∼1.7 fold higher than CD4^+^ T cell. Then, we examined, whether CD8^+^ T cells alone without the direct contact with CD4^+^ could have the same capacity. When CD4^+^ T cells were allowed to culture in inserts, so that there was no direct contact between CD8^+^ and CD4^+^ T cells, there was decreased production of both perforin (7.3% v/s 15.8%) and granzyme B (9.5% v/s 24.9%) producing CD8^+^ T cells (Fig. 4A). So, a direct contact of CD4^+^ and CD8^+^ T cells was necessary for sufficient production of cytolytic proteins. When we examined whether exogenous IL-2 could substitute the action of CD4^+^ T cells, we found that addition of 50 U/ml of IL-2 (excess amount) to CD8^+^ T cells, could produce both perforin and granzyme B equivalent to that of CD8^+^ T cells co-cultured with LipoK stimulated, *M. leprae* infected DCs in the presence of CD4^+^ T cells. However such high levels of IL-2 cannot be produced from host cells, in our experimental setting.

The intracellular level of another cytolytic protein, granulysin, was then examined. Enhancement of granulysin producing CD8^+^ T cells was observed when co-cultured with DCs activated with *M. leprae* and LipoK. As seen in Fig. 4C, 18.9% of activated CD8^high^ T cells and 28.4% of activated CD4^high^ T cells produced granulysin when co-cultured with DCs activated with *M. leprae* and LipoK, in comparison to those co-cultured with DCs activated with *M. leprae*, (1.7% of CD8^high^ T cells and 0.6% of CD4^high^ T cells).

### *Mycobacterium leprae* components were observed at the periphery of the infected DCs stimulated with LipoK, and co-cultured with T cells

**Figure 5:**
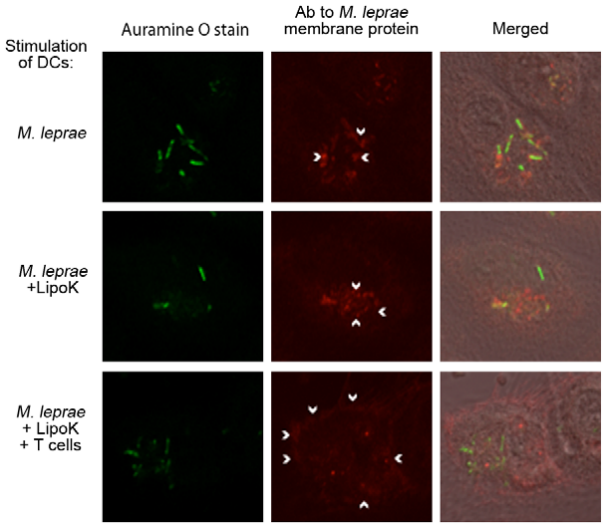
Localization of the membrane components of *M. leprae* at the periphery of the DCs. DCs were infected with either *M. leprae* alone or further stimulated with LipoK for 2 days and in some cases co-cultured with T cells. After 3 days co-culture with T cells, cover glass with attached DCs were fixed and observed under confocal microscopy-LSM5 Exciter. *M. leprae* was stained with Auramine O (shown in green) and *M. leprae* membrane components were stained with polyclonal rabbit antibody raised against the membrane fraction of *M. leprae* (depicted in red fluorescence). Alexa Fluor 633 conjugated anti-rabbit antibody (Molecular Probes) was used as the secondary antibody. Arrowheads indicate the positively stained region. Experiments were performed twice with different donors.

To examine the fate of *M. leprae* in activated DCs, the cells were stained with anti-*M. leprae* membrane polyclonal antibody. Confocal microscopy revealed rod shaped *M. leprae* as observed by auramine-O stain, and membrane components seem to be rather localized in the region where *M. leprae* are present (Fig. 5). Strickingly, those DCs stimulated with LipoK for 48 hours and co-cultured with T cells for additional 3 days showed membrane staining at the periphery of the DCs (Fig. 5 arrowheads shown), probably due to processing of the bacilli in activated DCs.

### Killing of *M. leprae* in DCs, by the LipoK stimulation

**Figure 6:**
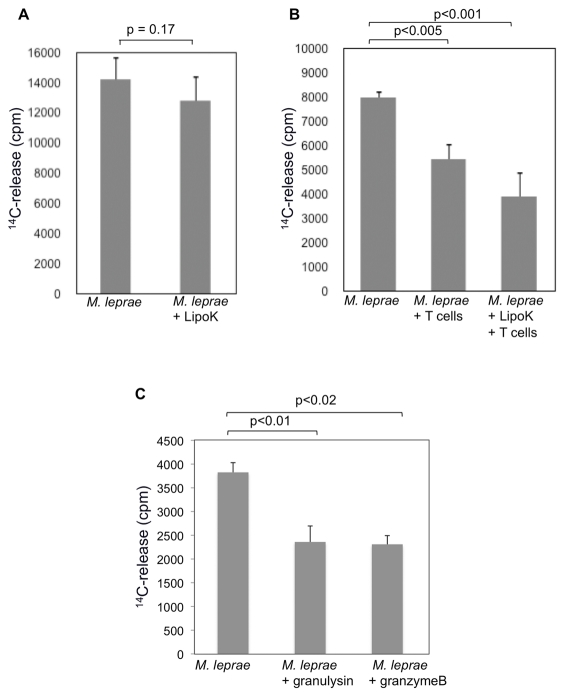
Reduction in the viability of *M. leprae* in DCs after co-culture with T cells and LipoK stimulation. (**A**) DCs were infected with *M. leprae* and stimulated with LipoK, 2 days later, cells were collected and the viability of *M. leprae* in DCs was measured by the radiorespirometric assay (metabolic CO_2_ release) as described in Materials and Methods. In brief, ^14^C labeled palmitic acid was added to the lysates of DCs and cultured at 33°C. After 7 days of culture, the amount of ^14^CO_2_ evolved was measured using a Packard 1500 TRI-CARB liquid scintillation analyzer. (**B**) DCs were infected with *M. leprae* as in **A**, and co-cultured with T cells. Six days after the co-culture, DCs were lysed, and the viability of *M. leprae* was determined by the radiorespirometric assay. (**C**) *M. leprae* at a concentration of 1×10^7^/well/200 µl in Middlebrook 7H9 media was incubated with granulysin or granzyme B for a period of 3 days at 33°C, and the viability determined as described in **A**. Unpaired Student's t test was used to find the statistical significance of the two sets of data. Representative data of three separate experiments is shown.

We determined the viability of *M. leprae* in DCs after stimulation with LipoK in the presence of autologous CD4^+^ and CD8^+^ T cells. Since *M. leprae* is uncultivable *in vitro*, the viability of *M. leprae* in DCs, after co-culture with the T cells for a week, was determined by the radiorespirometric assay. The amount of radioactive CO_2_ evolved which reflects the rate of ^14^C-palmitic acid oxidized by *M. leprae*, was measured by the scintillation counter. No significant reduction in ^14^CO_2_ production was observed, from DCs, not co-cultured with T cells, even in the presence of LipoK stimulation (Fig. 6A). But, when the bacilli were recovered from DCs stimulated with LipoK and co-cultured with T cells, ^14^CO_2_ production were significantly lower (p<0.001) than those recovered from DCs not stimulated with LipoK or T cells. The result indicates that approximately 50% reduction in the viability of *M. leprae* was observed in LipoK activated DCs and co-cultured with T cells compared to those obtained from DCs not stimulated with LipoK (Fig. 6B), indicating that T cells were essential and LipoK stimulation to DCs, was necessary to kill *M. leprae* in DCs. To further determine whether the cytolytic granules namely, granulysin and granzyme B could directly kill *M. leprae*, the bacilli was incubated with human granulysin or granzyme B for a period of 3 days at 33°C. Statistically significant reduction of ^14^CO_2_ was observed when the bacilli were incubated with granulysin as well as granzyme B (Fig. 6C).

## Discussion

In the present study we investigated the role of *M. leprae*-derived synthetic lipopeptide (LipoK), which consists of N-terminal 13 amino acids of the 33-kD *M. leprae* lipoprotein (Accession no. ML0603) linked to Palmitoyl-Cys((RS)-2,3-di(palmitoyloxy)-propyl group in the induction of intracellular killing of *M. leprae* through immuno-activation. Previously, we observed that the 33-kD lipoprotein and the truncated form of the protein induced the production of IL-12 in human peripheral blood monocytes [4], [5]. Although human DCs are potent inducers of acquired immune responses, when DCs were exposed to *M. leprae*, they are inefficient in activating T cells [21], [26]. It is generally recognized that, stimulation of T cells by intracellular pathogens, such as mycobacteria, is achieved by the coordinated processing of the antigens in the phago-lysosome of APCs and the expression of the antigenic determinants on APCs. Furthermore, CD40-CD40L interaction on immature DCs, are known to contribute to cell mediated responses in leprosy [27], [28]. In fact, when *M. leprae* infected DCs were stimulated with CD40L, up-regulation of CD83 and CD86 molecules was observed (not shown). However, we found that CD40L failed to induce the production of IL-12 p70 in *M. leprae* infected DCs. In contrast to CD40L stimulation, LipoK stimulation on *M. leprae* infected DCs induced significant production of IL-12. Further, the expression of CD40 on DCs was not enhanced by stimulating *M. leprae* infected DCs with LipoK. It was evident that IL-12 inducing ability of these matured DCs was mediated by TLR2, and not by other receptors such as mannose receptor or DC-SIGN, as observed in DCs exposed to *M. tuberculosis* or *M. bovis* BCG [29], [30], [31]. The TLR2 antagonistic antibody could almost totally inhibit the IL-12 production from DCs, as well as the T cell activating function of DCs (not shown), probably through blocking of the classical NF-kB pathway. Indeed, parthenolide, one of the major sesquiterpene lactones, known to inhibit NF-kB activity [24], inhibited the IL-12 production from DCs stimulated with *M. leprae* and LipoK. Also, IL-12 was efficiently produced when *M. leprae* was viable and not dead. Thus, although the exact mechanisms remain to be elucidated, some cell surface molecules and secreted components of *M. leprae* are responsible for the production of IL-12, which further modulates type 1 T cell responses [32], [33].

A number of mechanisms are known to be involved in the clearance of intracellular bacteria, such as IFN-γ release, apoptosis induction of the macrophages and anti-microbial activity of CTL [12], [15]. Production of IFN-γ could boost the ability to kill pathogens in host cells. In fact, it was found that LipoK activated *M. leprae* infected DCs, highly stimulated both memory CD4^+^ and CD8^+^ T cells, as well as naïve CD4^+^ to produce IFN-γ, and further assisted in the proliferation of both T cell subsets (Fig. 3). Inhibition of MHC class I and class II molecules on DCs, indicated that the activation of these T cells were MHC class II- and class I-dependent in CD4^+^ T cell and CD8^+^ T cells respectively. Further, proteolytic processing of *M. leprae* antigens was probably enhanced by LipoK treatment of DCs, since incubation with anti-*M. leprae* membrane Ab showed positive staining at the periphery of DCs, when co-cultured with T cells (Fig. 5). In addition, preliminary results showed that expression of MHC class I and II molecules on LipoK activated DCs, were elevated in those co-cultured with T cells. Thus, LipoK could probably assist in the processing and presentation of *M. leprae* antigens, and thereby, highly activate T cells.

The other important parameter, for the clearance of mycobacteria from the host cell, is their potential to activate antimicrobial effector mechanisms in human T cells. DCs have been shown to be involved in CTL induction following uptake of antigenic particles [25], [34], [35], [36]. CD8^+^ T cells co-cultured with LipoK stimulated *M. leprae*-infected DCs, through CD4^+^ T cells' help produced increased amount of cytolytic effector molecules: perforin and granzyme B. Adequate production of these cytolytic proteins from CD8^+^ T cells required direct contact with CD4^+^ T cells. Recently, there are studies that certain types of CD4^+^ T cells possess direct cytotoxic potential [25], [37], [38]. We observed a portion of CD4^high^ T cells (activated T cells), have the capacity to produce cytotoxic granules. Bastian et al. demonstrated that native *M. tuberculosis* heterogenous lipopeptides are potent immunogens for primary human T cells, and those T cells were CD4^+^ and MHC class II restricted, challenging the current concepts that cytotoxic T cells were restricted to CD8^+^ T cell subset [25]. Another lytic molecule, present in cytotoxic granules of T cells, is granulysin, which is reported to have direct anti-bacterial activity. Reports have shown the ability of T cells to secrete granulysin at the site of *M. leprae* infection, which provides evidence that anti-microbial activity of granule containing T cells is a mechanism of host defense in leprosy [39], [40]. We observed that LipoK stimulated, *M. leprae* infected DCs, highly enhanced the production of granulysin from CD8^+^ T cells. Unexpectedly, we observed that the percentage of CD4^+^ T cells producing granulysin was higher than CD8^+^ T cells. But, this fact was in lines with the earlier data, which showed co-localization of granulysin and CD4^+^ T cells in tuberculoid leprosy lesions [39], [40]. Thus, granulysin release by LipoK-mediated activation process, may lead to a direct anti-microbial effector pathway of host defense. These data demonstrated that both CD4^+^ T cells and CD8^+^ T cells, contribute to the induction of intracellular killing of *M. leprae*. These speculations were further supported by the fact that 50% of the phagocytosed bacilli were killed when infected DCs stimulated with LipoK, were co-cultured with T cells. This is the first observation of killing of *M. leprae* in an *ex vivo* system using human DCs and T cells. To further provide evidence of the effector mechanism at work during *M. leprae* killing by CTL, the direct effect of granulysin on *M. leprae* killing *in vitro* was analyzed. Results indicated that about 40% of *M. leprae* was killed by granulysin. Granulysin could probably lyse *M. leprae* by binding to the lipidic cell wall, through the same mechanism by which *M. tuberculosis* is destroyed by granulysin. Since, perforin is an essential molecule in the killing of intracellular *M. tuberculosis* [16], similar operation may be involved in intracellular *M. leprae* killing since perforin was effectively produced by T cells in our CTL culture system. On the other hand, direct killing of mycobacteria by granzymes is not known. But the viability of *M. leprae* was significantly lowered by granzyme B. Since granzyme B is one of the serine proteases that can target cytosolic and nuclear substrates to induce host cell death through mitochondrial perturbation, it may be involved in destroying the cell wall architecture of *M. leprae* by still unknown mechanism [41], [42]. The contribution of the cytotoxic granules to killing of bacteria remains to be of interest for further investigation. Together, the results indicate that LipoK could contribute to protective host response against leprosy and eventually kill the bacteria, through the production of perforin, granulysin and granzyme B in T cells.
